# Climate change and child health

**DOI:** 10.1111/jpc.16281

**Published:** 2022-12-03

**Authors:** Forbes McGain

**Affiliations:** ^1^ Departments of Anaesthetics and Intensive Care Western Health Melbourne Victoria Australia; ^2^ Department of Critical Care Medicine University of Melbourne Melbourne Victoria Australia

As another year of COVID‐19 passes by, I applaud the efforts of the editorial team of the Journal of Paediatrics and Child Health (JPCH) in devoting the November 2021 issue to climate change and child health.^1^ As an anaesthetist and ICU physician, I found Skowno and Weatherall's article^2^ most pertinent; please, no desflurane or laughing gas (N_2_O) anymore from any anaesthetists! Calls to greater sustainability action within the health‐care system by Kiang and Behne^3^ begin the march for the greater paediatric community. Quilty and Jupurrurla give insight into shared Indigenous/non‐Indigenous stories about climate change.^4^ Skinner's photograph of plastic detritus from one child's hospital lunch^5^ is a metaphor for *This Civilisation is Finished*.^6^ We must guide civilisation safely through the bottleneck of climate change and species mass extinction, driven by the twin challenges of over‐consumption and over‐population.

Yet guiding this sustainability journey requires enlightenment about our individual and communal carbon footprints from health care.^7^, ^8^ As an outsider to paediatrics, but someone immersed in hospital sustainability, I would like to comment about grappling with home and work carbon hotspots, and reference the work of Doctors for the Environment Australia (DEA),^9^ and the Climate and Health Alliance.^10^ Brace yourselves; some efforts are controversial, but should not be in 2022. This is not rocket science, but changing human behaviour is challenging.

## Simple: The Individual Paediatrician at Home

1. *Meat's a treat, please, less than once per week*. All plant proteins have <1/10th of the CO_2_e emissions of red meat.
2. Here's the elephant in the room: each child we bring into the world in a high‐income country will have an annual carbon footprint similar to yourself; so two is fine. Avoiding larger families will have an effect now (contrary to Dr. Skinner's views, although at least he mentioned it).^5^
3. No business class air travel (including conferences) as even one international Sydney‐Europe return flight (>10 t CO_2_e emissions) is close to the carbon emissions of *everything* else you (as an Australian) will do for the year.
4. So, yes, do instal solar panels, get off fossil/natural gas, insulate the house, double glaze your windows, ride your bike, buy an electric car, go holiday camping locally, but if you keep flying annually internationally then they are all complacent indulgences.^5^

## Intermediate: The Individual Paediatrician at Work

1. Bring indirect environmental sustainability into your everyday quality improvement and public health efforts at work. Such efforts include: antibiotic stewardship, Choosing Wisely, reducing waste, earlier safe home discharges from hospital, encouraging vaccinations, mentioning social determinants of health, clarifying why parents are smoking/drinking/etc, and assisting them to cease.
2. Focus on relevant hotspots. Even 10 min' N_2_O sedation in the Paediatric ED is equivalent to driving 150 km in a standard car. Each puff of a Ventolin metered dose inhaler (MDI, delivered with the high greenhouse warming propellant HFA 227ea) is like driving 1 km.^11^ Alternatives are possible (other sedatives replacing N_2_O, dry powder inhalers replacing MDIs).
3. Educate junior doctors, nurses on ward rounds and so forth, and bring environmental sustainability into the fold; talk about sustainability at least sometimes rather than overseas holidays or airline status points.
4. Write to your hospital encouraging them to mandate energy and procurement policies that ask suppliers to achieve improved environmental sustainability.
5. Augment the environmental sustainability of your private paediatric practice, learning from similar efforts by general practitioners.^12^

## Challenging: Collaborating at Work

Work matters more than home! Consider that a mid‐sized (300‐bed) hospital consumes the equivalent of 5000 houses worth of energy and, as individuals in hospitals, we create far greater waste due to ubiquitous single‐use items. What are the carbon hotspots that require prolonged action from multiple paediatricians collaborating with other physicians, nurses and administrators?

1. Go renewable, transition electricity away from fossil fuels. NZ, Tasmania, the ACT and South Australia are already largely there, and Victorian public hospitals will be there by 2025, so focus on Queensland and NSW.
2. Renewable electricity makes reusable equipment better,^9^ that is, as we move from fossil fuels to renewables for electricity to clean and sterilise reusable medical equipment, the carbon balance shifts in favour of reusable equipment.^13^
3. We need to quickly get off fossil gas, ensuring no new hospitals are built with gas heating, as has been shown to be possible in Canberra and Adelaide.^9^
4. Procure for a more environmentally sustainable future within hospitals and beyond (state health departments). The majority of health‐care's carbon (and financial) footprint stems from pharmaceuticals, equipment, imaging and pathology.^7^, ^8^
5. Advocate with the Doctors for the Environment Australia (DEA), Climate and Health Alliance (CAHA) and other bodies for a more environmentally sustainable health‐care system.

The *Race to Zero* is well and truly on.^14^ It is necessary to win it for our children.
